# The Limitations in Current Studies of Organic Fouling and Future Prospects

**DOI:** 10.3390/membranes11120922

**Published:** 2021-11-25

**Authors:** Xianghao Meng, Shujuan Meng, Yu Liu

**Affiliations:** 1 School of Space and Environment, Beihang University, Beijing 100191, China; sy1930212@buaa.edu.cn; 2 School of Civil and Environmental Engineering, Nanyang Technological University, 50 Nanyang Avenue, Singapore 639798, Singapore; cyliu@ntu.edu.sg

**Keywords:** membrane fouling, polysaccharide foulant, surrogate of foulants, fouling mechanism models

## Abstract

Microfiltration and ultrafiltration for water/wastewater treatment have gained global attention due to their high separation efficiency, while membrane fouling still remains one of their bottlenecks. In such a situation, many researchers attempt to obtain a deep understanding of fouling mechanisms and to develop effective fouling controls. Therefore, this article intends to trigger discussions on the appropriate choice of foulant surrogates and the application of mathematic models to analyze fouling mechanisms in these filtration processes. It has been found that the commonly used foulant surrogate (sodium alginate) cannot ideally represent the organic foulants in practical feed water to explore the fouling mechanisms. More surrogate foulants or extracellular polymeric substance (EPS) extracted from practical source water may be more suitable for use in the studies of membrane fouling problems. On the other hand, the support vector machine (SVM) which focuses on the general trends of filtration data may work as a more powerful simulation tool than traditional empirical models to predict complex filtration behaviors. Careful selection of foulant surrogate substances and the application of accurate mathematical modeling for fouling mechanisms would provide deep insights into the fouling problems.

## 1. Introduction

Wastewater reclamation is one of the effective countermeasures to tackle the water resource crisis. As a promising technology, membrane processes either via direct filtration or combined with other techniques have been widely used in the field of water treatment [1,2]. Membrane technology is highly efficient for organics removal and microbial interception. However, membrane fouling, which reduces the treatment efficiency and lifetime of membrane modules, is still a critical bottleneck restricting the wider development of membrane technology [3]. Membrane fouling refers to the phenomenon that sludge flocs, colloidal particles, bacteria/microorganism, dissolved macromolecules of organic matter or inorganic salts deposit on the membrane surface or are adsorbed in the membrane pores due to physicochemical and mechanical interactions with the membrane, resulting in a reduction or blockage in the membrane pores and irreversible decrease in membrane flux [4]. Current research interests in membrane fouling mainly include the following aspects: (i) membrane fabrication and modification for efficient fouling control; (ii) optimization of the operating conditions to minimize fouling (including the pre-treatment methods); (iii) adjustment of the environmental conditions such as the pH, presence of cations, and feed composition; and (iv) the development of novel simulation models for the analysis of fouling mechanism and prediction of fouling. All these foregoing studies pursue the same objective, i.e., fouling control, while the fundamental understanding of the fouling mechanism and the microstructure of fouling layers are still unclear. Therefore, this article attempts to illustrate the current challenging problems in understanding the fouling mechanism of ultrafiltration/microfiltration and try to provide more useful recommendations for fouling analysis and prevention.

## 2. Alginate: A Good Model for Organic Fouling Studies but Not Perfect

According to the type of dominant foulants, membrane fouling can be divided into four categories: organic fouling, inorganic fouling, biofouling, and colloid fouling [5]. Generally, fouling controls include the optimization of hydrodynamic conditions and operation parameters, online cleaning with backwash or chemicals, and ultrasonic cleaning, which were carefully selected according to the fouling types [6,7]. It has been found that the physico-chemical properties of the organic foulants in feed water play a significant role in determining its fouling propensity [8,9,10], which actually is the footstone for a fundamental understanding of the fouling mechanism. Furthermore, the complicated structure of biofilm formed on membrane surface is also deeply affected by the organic matters from feed water or secreted by the microorganisms [11]. The investigation on foulants identification and its molecular structure is crucial for fouling studies in the membrane related field. Therefore, researchers have made many efforts to decode the fouling mechanisms of organic foulants, trying to provide an effective solution to the fouling problem. The main organic foulants are protein, humic acid, and polysaccharides. Among them, polysaccharide foulants can cross-link or combine with other organic molecules to form a three-dimensional network structure, maintaining the mechanical stability of the foulant layer and thus playing a more important role than other organic foulants in membrane fouling [12,13]. Furthermore, the molecular structures of polysaccharide foulants rather than their function groups may play a more important role in membrane fouling [4,14]. In addition, some of the acidic polysaccharide foulants form transparent exopolymer particles (TEP), especially in the presence of divalent and trivalent cations, which influence the fouling development including organic fouling and biofouling [11,15]. These findings contribute to a better understanding of the fouling mechanisms and promote the development of the techniques in mitigating fouling problems.

In order to achieve a fundamental understanding of the fouling mechanism, surrogate foulant is usually employed in fouling studies. In 2002, alginate was considered to have the representative function of extracellular polymeric substance (EPS) in biofilms by fluorescence labeling [16]. Since then, sodium alginate was widely employed in studies of organic fouling and biofouling to represent the polysaccharide foulants or even organic matter [16,17,18]. However, some researchers have claimed that sodium alginate was a poor representation for either natural organic matter or organic matter in effluent by comparing the organic composition [19]. Whereafter, Yang et al. also reported the substantial differences in the colloid properties and membrane fouling behaviors between sodium alginate and EPS [20]. As a consequence of these findings, employing sodium alginate to represent all polysaccharides in fouling studies is not perfect. In our previous research, we investigated seven common polysaccharides and tried to find their differences in fouling mechanisms [4]. Combined with model simulation, we classified them into four fouling models. This attempt shows the great divergence lying in the abundant organic foulants that can be present in the various feed water to membrane systems. Thus, this “one-size-fits-all” approach is insufficient to deal with the complexity of the organic foulant found in feed water to membrane systems. Generally, the feed waters to membrane systems in water treatment include three types: surface water for water supply, effluent from wastewater treatment plants (WWTP), and seawater for desalination. EPS contains more organic matter. If membrane fouling is carried out according to these three kinds of extraction, it will be more representative and more targeted. In view of this, more surrogate foulants or EPS extracted from practical feed water should be applied to the studies of membrane fouling problems.

## 3. Advances in Modeling of Membrane Fouling: Traditional versus Nontraditional Approaches

Constructing a mathematic model is a good way to analyze membrane fouling. A fouling model can provide a qualitative evaluation of fouling type, figure out the main cause of fouling, and further predict the fouling potential of a certain feed. With the wide application of membrane separation technology, many filtration models have been established, such as artificial neural network (ANN), genetic programming (GP) models, variance partitioning analysis (VPA), and the classic Hermia models [21,22,23,24]. As one of the state-of-the-art techniques, ANN possesses the ability of nonlinear function approach and self-adaptive ability [25]. However, ANN requires a large number of parameters (such as network topology, the initial value of weight and threshold, etc.) and the speed of network learning and the detection accuracy is subject to the number of training samples [25]. It is difficult to obtain a large database of filtration data; as such, this model cannot reach a high degree of accuracy. In addition, GP was proposed as a novel approach in modeling membrane fouling to predict permeate flux. Similar limitations with the ANN approach are present in this GP model.

Certainly, the mathematical models mentioned above explain the mechanisms of the membrane filtration process to a certain extent. However, these mathematical models are extremely complex with many parameters and assumptions, which usually lead to difficulty in parameter estimation and limited application scope [26,27]. Hermia established four classical filtration models based on different fouling types, which depend on the value of n in Equations (1) and (2) [15,28,29].

(1) $$\frac{d^{2}t}{dV^{2}}=K{\left[\frac{dt}{dV}\right]}^{n}$$

(2) $$\frac{dJ}{dt}=-KJ{\left(A_{eff}J\right)}^{2-n}$$

*K* is a constant dependent on the property of membrane, *A_eff_* is the effective surface area of membrane (m^2^), and n is the blocking index dependent on the mechanism of membrane fouling. When *n* equals 2, 1.5, 1, or 0, Equation (2) can be simplified as complete blocking, intermediate blocking, standard blocking, and cake layer. The Hermia model used the filtration data (*J*/*J_o_*, i.e., the decline percentage of permeate flux) to predict the fouling mechanisms. These models mainly take into account fouling trends, which are “filtration data” in the paper. However, there are too many assumptions in the derivation of the mathematic model, and this mechanical partitioning method makes the model’s criticality unclear. Meanwhile, the classic Hermia models are established for specific operation modes, while some inappropriate applications occur due to a misunderstanding of these complex equations. Some endeavors have been made to improve the accuracy of the Hermia models. For example, the applicable scope of Hermia classical model and mass transfer model is clarified by investigating the role of structural and functional features of various polysaccharides in membrane fouling [4]. However, this approach is time-consuming and not applicable to all cases. Moreover, the Hermia models assume four ideal scenarios which are not always in accordance with the practical filtration conditions [4]. In the practical processes of membrane filtration, many factors have an impact on the results of the model fitting including membrane characteristics, feed properties and filtration modes [12]. More importantly, most of the existing models are based on laboratory data (such as resistance-in-series model and Hermia models) rather than field data. It has a positive effect on the study of the adsorption of foulants on the membrane surface but reduces the reliability of the model applied to practical systems. Therefore, in order to better control fouling problems, an effective model combining the mechanism analysis with the empirical model should be established to provide decision support for the optimal operation of membrane systems.

The support vector machine (SVM) based on structural risk minimization is a machine learning method suitable for small-scale datasets, which shows excellent performance in limited samples, non-linear function, and multidimensional pattern recognition. SVM has been applied to predict the protein types by employing the composition of amino acids as inputs, which clearly shows the above advantages of SVM in machine learning [30]. Therefore, we consider identifying and establishing the connection between input parameters (filtration data) and output parameters (fouling mechanism) by SVM to predict complex filtration processes. SVM was considered as a reliable accurate estimation method to assess the soil quality, screen the non-target contaminants, and predict the adsorption performance for application in the environmental field [31,32,33]. The employment of SVM to validate membrane fouling was firstly proposed in the 2007 second IEEE Conference on Industrial Electronics and Applications. In this study, it mainly considers three influential factors of membrane fouling, including the property of membrane, operation condition and the characteristic of filtrate. Recently, researchers found that the SVM technique outperforms the other models in estimating the fouling resistance in MBR processes [34]. Therefore, the application analysis of SVM in membrane fouling is presented from this perspective. The detailed operation procedure of SVM mainly includes the following steps: Firstly, the collection of sample data is considered to be the primary task for the evaluation of membrane fouling using SVM. The characteristic parameters of membrane fouling in the sample data are then screened to adapt to different operating conditions. Based on this, the key parameters are determined by optimizing the screening. All characteristic parameters which reflect the degree of membrane fouling could be obtained and selected by the forward model. For example, eight characteristic parameters such as initial membrane flux were chosen as input vectors of SVM and adopted as the radial basis function to improve the degree of accuracy by training and testing samples [25]. Based on the full parameterization optimize, SVM can be used to focus on one of the multi-parameters, i.e., filtration behaviors. Although the operating pressure, pumping time, and other characteristic parameters are simplified to a certain extent, the fouling mechanism can be more accurately identified and predicted. To obtain general trends in the files of membrane filtration, the weight of membrane flux to describe the whole process of membrane fouling should be increased, and other operation parameters should be simplified. As such, the database of SVM training based on mathematical models and practical parameters can be obtained to identify the fouling mechanisms of unknown feed water to the membrane system. Thus, due to the nonlinear relationship between the operating variables and the output product of the fouling process, SVM may be much more powerful than traditional empirical models to predict complex filtration processes. 

## 4. Remarks

In order to obtain a fundamental understanding of membrane fouling, it is crucial to establish a detailed database of reference foulants including important categories. Mathematical models can provide an insight into the fouling mechanism, while the present models are limited in accurately characterizing the complex membrane fouling problems. SVM may work as a good tool to recognize the fouling mechanisms and seek an appropriate surrogate for foulants in practical feed water. Consequently, with the successful establishment of a database including representative foulants and the improvement of mathematical models in predicting fouling, the fouling problems can be well understood and effectively controlled.

## Data Availability

All the data supporting the findings of this study are available within the article.
